# Can phenotypic rescue from harvest refuges buffer wild sheep from selective hunting?

**DOI:** 10.1002/ece3.1185

**Published:** 2014-08-14

**Authors:** Fanie Pelletier, Marco Festa-Bianchet, Jon T Jorgenson, Chiarastella Feder, Anne Hubbs

**Affiliations:** 1 Département de biologie, Université de Sherbrooke2500 boulevard de l'université, Sherbrooke, Quebec, J1K 2R1, Canada; 2 Alberta Department of Sustainable Resource DevelopmentSuite 201, 800 Railway Ave., Canmore, Alberta, T1W 1P1, Canada; 3 Fish and Wildlife Division, Alberta Department of Sustainable Resource Development4919-51st St., Rocky Mountain House, Alberta, T4T 1B3, Canada

**Keywords:** Artificial selection, harvest, *Ovis canadensis*, parks, source-sink dynamics, trophy hunting, ungulates

## Abstract

Human harvests can unwittingly drive evolution on morphology and life history, and these selective effects may be detrimental to the management of natural resources. Although theory suggests that harvest refuges, as sources of unselected animals, could buffer the effects of human exploitation on wild populations, few studies have assessed their efficiency. We analyzed records from >7000 trophy bighorn rams (*Ovis canadensis*) harvested in Alberta, Canada, between 1974 and 2011 to investigate if the movement of rams from refuges toward harvested areas reduced the effects of selective harvesting on horn size through phenotypic rescue. Rams taken near refuges had horns on average about 3% longer than rams shot far from refuges and were slightly older, suggesting migration from refuges into hunted areas. Rams from areas adjacent to and far from harvest refuges, however, showed similar declines in horn length and increases in age at harvest over time, indicating a decreasing rate of horn growth. Our study suggests that the influx of rams from refuges is not sufficient to mitigate the selective effects of sheep trophy harvest. Instead, we suggest that selective hunting of highly mobile animals may affect the genetic structure of populations that spend part of the year inside protected areas.

## Introduction

Human harvests can have important ecological and evolutionary consequences (Milner et al. 2007; Allendorf and Hard 2009). Organisms subject to consistent and strong selective harvesting, that target specific heritable characteristics such as tusks, antlers, horn, or body size, may respond to these new artificial selective pressures (Darimont et al. 2009). For example, over the last century, intense poaching of African elephants (*Loxodonta africana*) for the illegal ivory trade led to an increase in the proportion of tuskless females (Jachmann et al. 1995). Evolutionary effects in harvested species include dwarfing of Himalayan snow lotus (*Saussurea laniceps*) (Law and Salick 2005), reduction in size at maturity of fish (Olsen et al. 2004; Hutchings 2009) and of fighting conch (*Strombus pugilis*) (O'Dea et al. 2014) and changes in size and shape of horns in trophy-hunted ungulates (Coltman et al. 2003; Garel et al. 2007; Pérez et al. 2011). Recent meta-analyses revealed that selective pressures on wild species arising from human activities are typically higher than those caused by natural drivers, leading to higher rates of phenotypic change in size or life-history traits (Hendry et al. 2008; Darimont et al. 2009). In addition, these changes are not necessarily rapidly reversed by natural selection when artificial selection ceases, because natural selective pressures are typically much weaker than artificial ones (Conover et al. 2009). Together, these studies provide strong evidence that exploitation can unwittingly drive evolution (Allendorf and Hard 2009). Therefore, these potential ecological and evolutionary impacts must be considered when managing natural resources (Stockwell et al. 2003; Kinnison et al. 2007).

Several theoretical studies have suggested that protected areas, with no or reduced exploitation (hereafter named refuges) may reduce the ecological and evolutionary effects of selective harvesting in adjacent exploited areas (Tenhumberg et al. 2004; Baskett et al. 2005; Dunlop et al. 2009). These models assume that refuge populations are a source of unselected immigrants into harvested populations. Thus, in species where dispersal is sufficient, emigration from refuges into intensively harvested areas may counteract the phenotypic and genetic impacts of selective harvesting. For example, Baskett et al. (2005) suggested that marine reserves can reduce fisheries-induced selection for smaller sizes at maturation, if reserves are large relative to the target species' dispersal range.

Empirical studies assessing the effectiveness of refuges to mitigate the effects of selective harvesting, however, are scarce, particularly for terrestrial systems. That is partly because the required data on genotype and phenotype inside and outside refuges are not available. An alternative approach is to compare temporal trends in population dynamics, life history and morphology of populations located near and far from refuges. This approach recently revealed that the establishment of marine protected areas for shellfish increased the abundance of European lobster (*Homarus gammarus*) in nearby fishing areas by about 160% and lobster size by 13% (Moland et al. 2013). Similar effects on population density and body size were reported for Atlantic cod (*Gadus morhua*) (Moland et al. 2013). In Zimbabwe, horn size of harvested impala (*Aepyceros melampus*) decreased with distance from a national park, but the size of horns of sable antelope (*Hippotragus niger*) increased (Crosmary et al. 2014).

The goal of this study was to evaluate the potential for “phenotypic rescue”, defined as the migration of unselected rams from refuges to harvested areas, to mitigate the effect of trophy hunting on bighorn sheep (*Ovis canadensis*). Assuming that in protected populations males are older and larger than those in selectively hunted populations, if the influx of rams from refuges is sufficient, then we predicted that rams harvested in areas near refuges should be older and larger than rams harvested in areas further from refuges. Similarly, the decline in horn size over time should be shallower in areas with a possible influx of unselected rams. To test these hypotheses, we examined records of more than 7000 trophy rams harvested in Alberta, Canada, between 1974 and 2011. We compared horn size and age at harvest of males in hunting units adjacent to protected areas to those shot in units further away. The hunt begins in late August or early September and lasts until the end of October, about 3 weeks before the start of the rut (Festa-Bianchet et al. 2014). As males start searching for females during this period (Hogg 2000; Pelletier et al. 2006), rams from protected areas may move into hunted areas. We therefore investigated the effect of harvest date within a hunting season on horn size, to test the hypothesis that large-horned males exit protected areas and become available for harvest late in the season. We investigated whether temporal changes in horn size and age at harvest differed between areas adjacent to and far from harvest refuges. Over the last 37 years, horn size of harvested bighorn rams in Alberta has declined and age at harvest increased, suggesting slower horn growth rate (Pelletier et al. 2012; Festa-Bianchet et al. 2014). If dispersal of animals from refuges partly buffers the effect of selective hunting, we predicted that the age of rams shot near refuges would remain lower than for rams shot farther from refuges, as faster horn growth would allow rams to reach harvestable horn size at a younger age. Similarly, we predicted a steeper temporal decline in horn size for harvested rams in areas located far than near refuges.

## Materials and Methods

### Data collection

**Figure 1:**
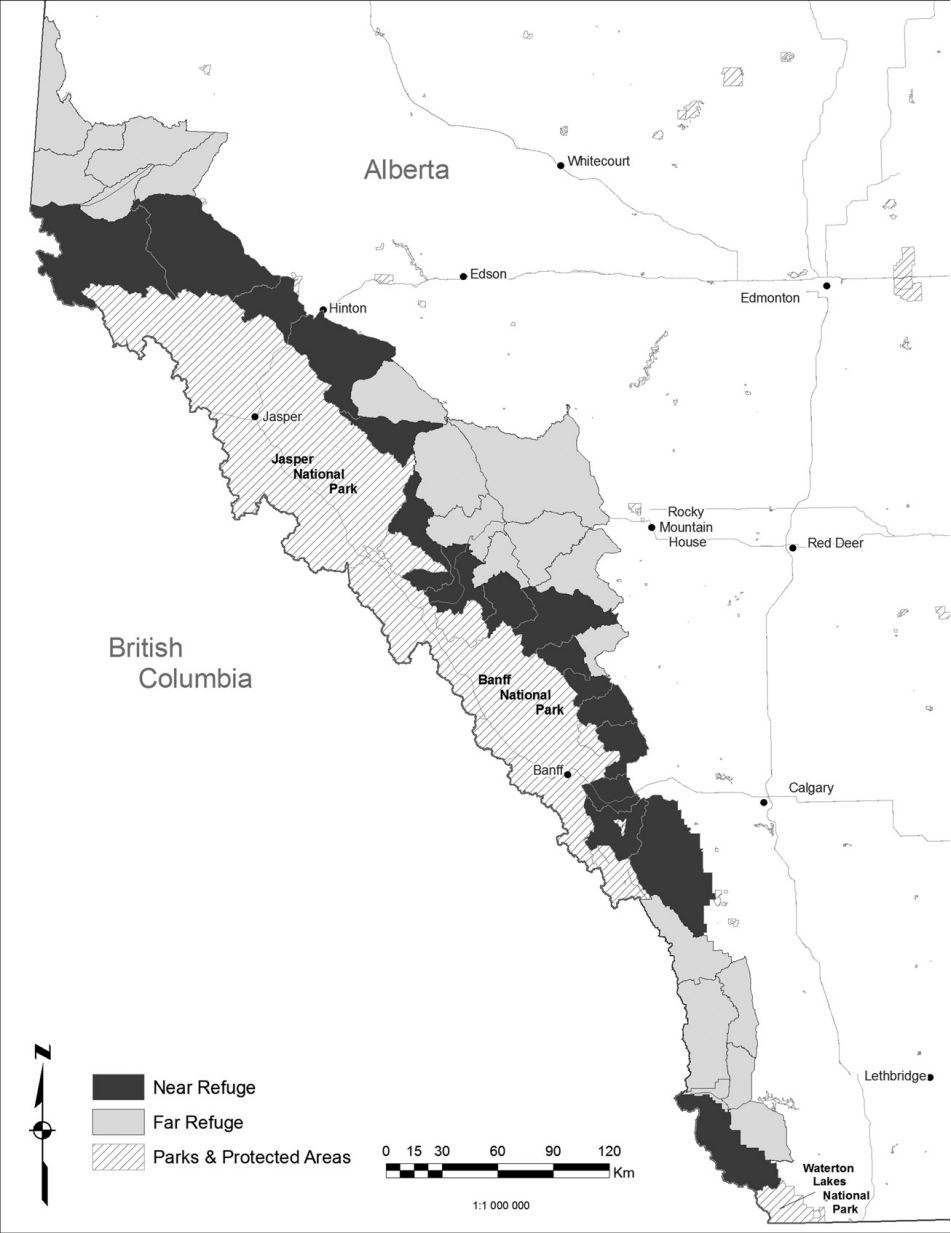
Map of Wildlife Management Units (WMU) where bighorn sheep are hunted and of hunting refuges in Alberta, Canada. The dark WMUs, adjacent to protected areas, were classified as “near hunting refuge”; light gray one were treated “as far” (see text).

We used information collected by wildlife management staff on more than 7000 harvested rams in Alberta, Canada, over 37 years (1974–2011). During this period, most populations of bighorn sheep outside protected areas were hunted under a regulation stipulating that a ram could be harvested if the tip of at least one horn surpassed a straight line drawn from the front of the base of the horn to the front of the eye when viewed in profile (Pelletier et al. 2012). Rams that fit this definition are referred to as “legal”. A “trophy sheep” license allows the killing of one legal ram during the hunting season. Any Alberta resident can purchase one “trophy sheep” license per year. About 80 additional licenses are available to nonresidents, who must engage a professional outfitter. Therefore, there are no limits on trophy ram harvests other than the availability of legal rams. Successful hunters must submit the head of harvested rams for compulsory inspection and measurement to Alberta Fish & Wildlife personnel. For each ram, officials record the age by counting the horn annuli and measure (cm) total length along the outside curvature and base circumference of both horns. They also note the Wildlife Management Unit (WMU) where the ram was harvested and whether or not the hunter is an Alberta resident. There are 41 WMUs in Alberta with a trophy sheep season. To test whether ram harvested near refuges were larger than ram harvested away from them, we assigned males shot in WMUs contiguous to a harvest refuge (mostly national parks) to a “near” category and males harvested in WMUs not contiguous to a refuge to a “far” category (Fig.1). Two WMUs (Fig.1) that share a short boundary with refuges were included in the far categories because local knowledge from wildlife managers suggests limited migration of rams from refuges into these WMUs. The harvest database was first checked by Alberta Fish & Wildlife biologists to remove entries with missing horn measurements, ram age, or obvious errors, such as harvest dates outside the hunting season. Illegally harvested rams (primarily sheep that did not meet the legal definition or were shot outside the hunting season) made up 1.7% of the data and were excluded from analyses. We excluded these rams because we use mean horn size of legal rams to compare populations. If we included poached rams which have not yet reached four-fifth of horn curl, areas with higher poaching rates could appear to produce smaller rams. We also excluded rams taken by First Nations, as subsistence harvest is not restricted by horn size nor based on licensing requirements. In a few areas, a full curl regulation was adopted in the late 1990s: Under this definition, to be “legal,” rams had to have longer horns than what we describe above. We excluded animal harvested in these areas under this regime from analyses. The final sample size included 5033 rams shot near refuges and 2054 rams shot far from refuges. Over the years of the study, the population of bighorn sheep in Alberta did not show any major temporal trend and was estimated at about 6000-7000 in provincial lands and 4000-4500 in National Parks (Jorgenson 2008).

### Statistical analyses

Age at death, horn length, and base circumference were analyzed using linear mixed effect models (Pinheiro and Bates 2000). Alberta Fish & Wildlife biologists have grouped WMUs with trophy sheep seasons into eight Sheep Management Areas (SMA), based on genetic differences and natural barriers to movement (Festa-Bianchet et al. 2014). We included Sheep Management Area as a random effect to account for both regional differences in horn size and changes in the distribution of the harvest over the years of the study. To test whether the decline in horn size and increase in age at death were less pronounced in areas near refuges, we included an interaction between harvest year and refuge (near vs far). As the temporal trends in horn size and age at harvest were nonlinear (Festa-Bianchet et al. 2014), we also included an interaction with harvest year^2^. The interaction between refuges and year^2^ was not significant and was excluded from final models. We included the average monthly values of the Pacific Decadal Oscillation from April to September (summer PDO) during the first 4 years of each ram's life (Loehr et al. 2010; Festa-Bianchet et al. 2014) to account for confounding effects of climate. Most horn growth occurs during the first 4 year of life (Bonenfant et al. 2009). As reported in Festa-Bianchet et al. (2014), the average summer PDO when rams were aged 1–4 years was associated with decreasing age at harvest and increasing base circumference but had no effect on horn length. Models of horn size also accounted for age at harvest. Horn size of harvested rams declined, while age at harvest increased over the last 30 years in both Alberta (Festa-Bianchet et al. 2014) and British Columbia (Hengeveld and Festa-Bianchet 2010). Finally, we calculated the prop-ortion of rams aged 4 and 5 years in the harvest, and we tested whether changes in age structure varied in areas near and far from refuges. At 4 or 5 years of age, only rams with rapid growth rates and the largest horns fit the definition of legal ram and may be harvested. This analysis used a general linear model, including harvest year, proximity to refuges, and an interaction between these variables.

All analyses began with a full model including all covariates, the interactions previously mentioned, and random effects. Then, we tested the significance of random effects with likelihood ratio tests, using restricted maximum likelihood (Pinheiro and Bates 2000). If random effects were not significant, we continued using linear or generalized linear models depending on the response variable. We then used backward selection to remove nonsignificant fixed effects (Crawley 2007). All analyses were implemented in R version 2.15 (R Development Core Team 2012). The “nlme” package was used to fit generalized mixed effects models.

## Results

**Figure 2:**
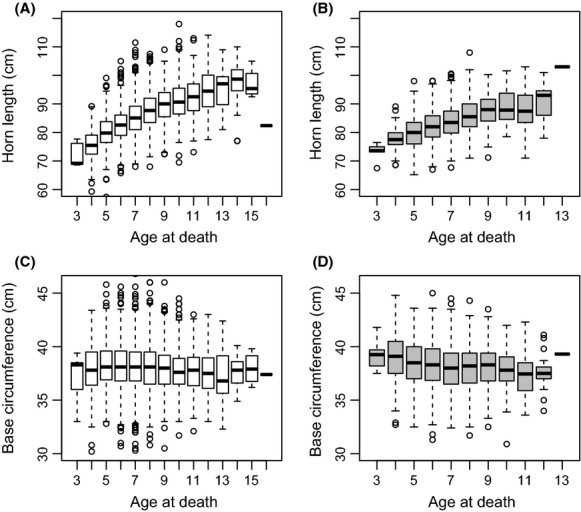
Box plots of unadjusted horn length and base circumference as a function of age for bighorn rams shot in hunting areas near (A, C) and far (B, D) from protected areas in 1974–2011 in Alberta, Canada. The box represents the 25th, median, and 75th percentiles of the raw data.

**Figure 3:**
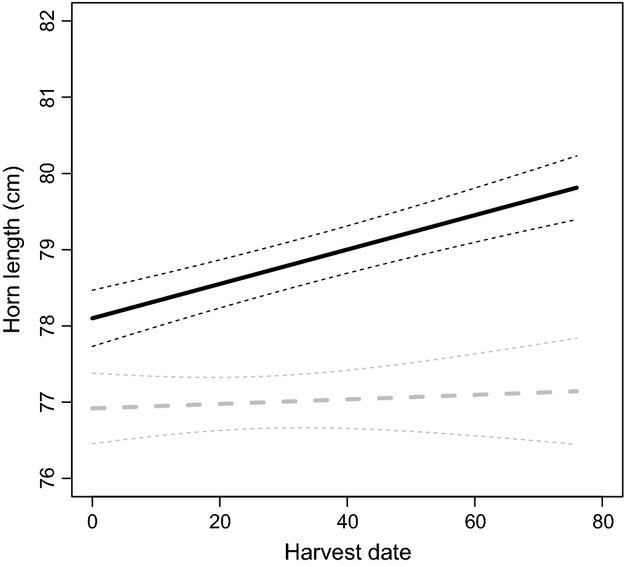
Combined effects of harvest date and refuge proximity on horn length (cm) with 95% confidence intervals of bighorn rams shot in hunting areas adjacent to (solid and black line) or far from (dashed and gray line) harvest refuges, 1974–2011, Alberta, Canada. For illustration purposes, we present the predicted line for 5-year-old rams.

The horns of rams harvested near refuges were on average about 3% longer than those of rams shot far from refuges (Fig.2A and B). This small difference was significant, and it increased slightly as the season progressed (Table1A, Fig.3). We found no effect of PDO on horn length (−0.262 ± 0.168, *t* = 1.552, *P* = 0.12). Horn base circumference was independent of location of harvest (average ± SD, cm: far = 38.2 ± 2.1, near = 38.1 ± 2.1, Table1B). There was no effect of harvest date on horn base circumference (0.002 ± 0.001, *t* = 1.518, *P* = 0.13), and although there was a trend for rams shot near refuges toward the end of the season to have slightly larger bases, this difference of less than 5 mm was not significant (0.0043 ± 0.002, *t* = 1.884, *P* = 0.06). Rams shot near refuges were significantly older than those shot further away, but this difference averaged only 0.35 years (Table1C). There was no effect of harvest date within the hunting season on ram age (−0.001 ± 0.009, *t* = 1.625, *P* = 0.10) and no interaction between refuge proximity and date of harvest on age (0.001 ± 0.002, *t* = 0.700, *P* = 0.48).

**Table 1:** Temporal trends in A) horn length (cm), B) horn base circumference (cm), and C) age at death (years) for bighorn rams shot in Alberta, 1974–2011. Estimates are from linear mixed effect models with Sheep Management Area as random effect. PDO is the average summer Pacific Decadal Oscillation, while rams were aged 1 to 4 years. Sample sizes differ as not all measurements were available for all rams. The reference category for refuge proximity is “far”, so that positive coefficients indicate a positive effect of being harvested near a refuge.

Variables	Coefficient	SE	*T*-value	*P*-value
(A) Horn Length				
Harvest year	19.541	2.655	4.571	<0.001
Harvest year^2^	−0.005	0.0007	7.369	<0.001
Age	4.574	0.217	21.068	<0.001
Age^2^	−0.155	0.014	11.323	<0.001
Refuge proximity	1.182	0.259	4.571	<0.001
Harvest date	0.003	0.006	0.501	0.617
Refuge proximity*harvest date	0.020	0.007	2.917	0.004
(B) Horn Base				
Harvest year	−0.008	0.002	3.287	0.001
Age	0.096	0.075	1.288	0.198
Age^2^	−0.009	0.005	1.986	0.047
PDO	0.242	0.039	6.159	<0.001
Refuge proximity	0.242	0.062	3.889	<0.001
(C) Age				
Harvest year	9.217	1.212	7.605	<0.001
Harvest year^2^	−0.002	0.0003	7.589	<0.001
PDO	−0.572	0.053	10.822	<0.001
Refuge proximity	0.349	0.057	6.141	<0.001

To explore whether the temporal decline in horn size was reduced near refuges compared with areas unlikely to benefit from phenotypic rescue, we tested for an interaction (represented by *) between harvest year and proximity to refuges affecting horn size. Temporal declines in horn length (harvest year * refuge proximity: 0.839 ± 6.028, *t* = 1.300, *P* = 0.19, harvest year^2^ * refuge proximity: −0.002 ± 0.002, *t* = 1.301, *P* = 0.19) and horn base circumference (harvest year * refuge proximity: 0.343 ± 2.070, *t* = 0.166, *P* = 0.87, harvest year^2^ * refuge proximity: −0.0001 ± 0.0005, *t* = 0.164, *P* = 0.87) did not differ near and far from refuges. Similarly, the age of rams shot near and far from refuges increased at similar rates (harvest year * refuge proximity: 2.937 ± 1.870, *t* = 1.571, *P* = 0.12, harvest year^2^ * refuge proximity: −0.001 ± 0.0005, t = −1.564, *P* = 0.12).

**Figure 4:**
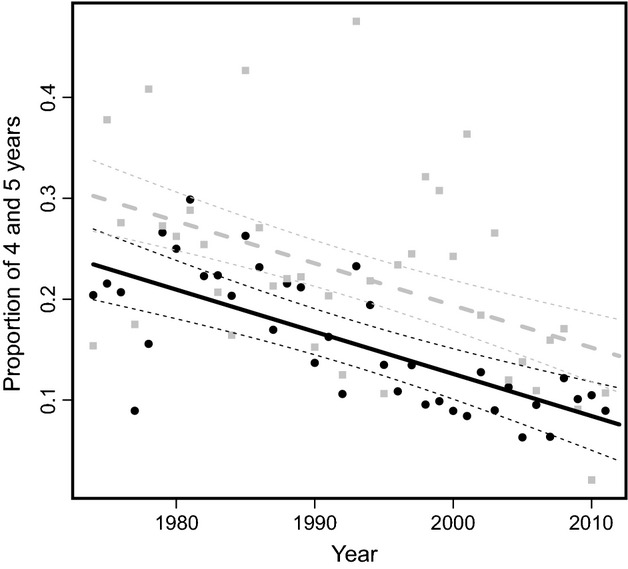
Proportion of 4- and 5-year-old rams harvested by year with 95% confidence intervals in areas located near (solid and black line) and far (dashed and gray line) from harvest refuges in 1974–2011 in Alberta, Canada.

The age structure of the harvest changed according to proximity to harvest refuges. More rams aged 4 and 5 years (23% of the harvest) were harvested far from than near refuges (16%) (−0.067 ± 0.016, *t* = −4.174, *P* < 0.001, Fig.4), but the rate of decline in the proportion of young rams in the harvest over time was the same in both areas (interaction between harvest year and refuge proximity: −0.0003 ± 0.001, *t* = 0.248, *P* = 0.80) which was counter to expectations.

## Discussion

Theoretical models suggest that refuges can buffer wild populations from selective effects of human exploitation (Baskett et al. 2005; Dunlop et al. 2009). Only a handful of studies have tested this hypothesis in the wild, and to our knowledge, almost all were in marine ecosystems (see for example, Moland et al. 2013). Our analyses suggest that although rams shot near harvest refuges are slightly larger and older, refuges did not buffer bighorn sheep from the selective effect of unlimited, phenotype-based trophy hunting because declines in horn growth and increases in age at harvest were similar regardless of how close hunting units were to the refuges.

The increase in horn size of rams harvested near refuges from late August to late October is consistent with the behavior of mature males that start prospecting for breeding ewes in October as the rut nears (Pelletier et al. 2006). At that time, rams may move over tens of kilometers (Festa-Bianchet 1986; Hogg 2000), and it seems likely that hunters harvest some rams moving out of protected areas. Earlier in the season, hunters mostly harvest resident rams that should be generally smaller than rams in protected areas because they are at risk of harvest as soon as their horns attain legal status. The small differences in age and size of harvested rams and the similar temporal trends for these traits in hunting areas near and far from refuges, however, suggest that dispersal from refuges occurs but is insufficient to buffer harvested population from artificial selection, possibly because most rams exiting refuges are shot before the rut. Unlike a recent study on a marine system (Moland et al. 2013), our results suggest that for bighorn sheep populations in Alberta, there is limited phenotypic rescue from refuges to hunted areas.

Our finding of larger animals shot nearer to refuges is similar to that for a recent study on impala (Crosmary et al. 2014). In our study, average horn length near and far from refuges, however, only differed by about 2.6%. Although the average horn length of rams harvested near refuges was 85.5 cm, many of the very largest rams harvested in the province were taken there: Average horn length of the largest 1% of rams was 104.3 cm near refuges, compared with 99.5 cm elsewhere. These exceptional rams were on average 1 year older near refuges (13 vs. 12 years). They did not, however, differ in horn base circumference (43.4 vs. 43.3 cm). This result is consistent with a scenario where a few large males occasionally exit refuges during the hunting season and are killed (Hogg 2000), but most rams harvested near refuges are of similar size and age to those taken elsewhere. The slight increase in horn size of rams harvested near refuges late in the season further supports the suggestion that some rams exit the refuges and are harvested as the rut approaches (Hogg 2000). It appears, however, that most of these rams are taken before they can develop large horns, as they would be subject to the same high hunting pressure as other rams in the hunted areas.

We observed similar temporal trends in areas near and far from refuges. Horn length, horn base circumference, and the proportion of 4- and 5-year-olds in the harvest declined, while the average age at harvest increased, independently of the proximity of harvest refuges. Sample size near refuges was higher than far from refuges (yearly averages of 132 and 54 rams, respectively), so yearly averages for areas far from refuges may be more affected by stochasticity. Overall, the substantial temporal decline in the proportion of young rams in the harvest suggests a slower rate of horn growth in recent years compared with a few decades ago (Festa-Bianchet et al. 2014). These results suggest that existing harvest refuges in Alberta (mostly Banff, Jasper and Waterton National Parks and Kananaskis Provincial Park, Fig.1), despite their large size and large populations of bighorn sheep (Jorgenson 2008), are insufficient to mitigate artificial selection in bighorn rams in hunted areas.

There are two mechanisms by which phenotypic rescue through source-sink dynamics of unselected phenotypes could mitigate the effect of selective hunting. First, if populations in refuges are large enough, emigrating large males could contribute to the yearly harvest. If those males were harvested before reproducing, then mitigation of decline in horn size in areas adjacent to refuges would mostly be due to phenotypic and/or demographic effect. If harvest rate were low, or if most males remained in refuges until after the hunting season, this source-sink dynamic could dampen the selective effect of the trophy hunt through gene flow because unselected males could reproduce in areas of high harvest rate where most of their competitors would have been shot before the rut (Tenhumberg et al. 2004; Baskett et al. 2005). Harvest data do not provide information on rams that may exit refuges after the hunting season to breed, but they show that although larger and older males are harvest near refuges, neither phenotypic nor evolutionary rescue is taking place, as we observe similar temporal trends in both types of areas.

Posthunt winter aerial surveys in central Alberta in 2011–2013 reported more legal rams inside refuges (about 34% of adult rams) than outside (about 21% of adult rams) (Alberta Fish & Wildlife, unpublished reports). Information on age-specific horn size of rams that remain inside refuges would be particularly valuable in this context, but is unavailable. Although we accounted for variation in climate, we are unable to completely reject the hypothesis that environmental change is causing the observed temporal trends, independently of a selective effect of phenotype-based harvest. The increase in average horn length of rams taken near refuges as the hunting season progressed was not accompanied by an increase in age at harvest, suggesting that rams taken in October had slightly larger horns for their age than those taken in late August and September. Given that horn growth should have stopped by October (Hoefs and Nette 1982) and that in areas far from refuges, horn length was not affected by harvest date, these results are consistent with a limited influx of rams with faster horn growth rate from populations not subjected to artificial selection through trophy hunting. As mentioned, whether or not these unselected rams contribute to an evolutionary rescue will then depend on their chance to survive to breed within the hunted populations.

Our study suggests that refuges may increase the number of rams harvested in nearby hunted areas, but do not buffer against temporal trends likely due to selective harvest. Our analysis also raises the question of the possible effects that trophy hunting may have on bighorn sheep populations inside protected areas. Hogg (2000) suggested that middle-ranking rams may move out of protected areas before the rut because their mating success may be higher in areas where many competitors will have been harvested. That suggestion implies a gene flow mostly from protected to hunted areas, possibly lowering genetic variability inside refuges. If the larger, top-ranking rams stay within refuges to mate, however, they would not contribute to phenotypic rescue of adjacent populations. More precise information on the dates of breeding migrations, the age structure and proportion of rams leaving, the survival of rams that exit refuges before the rut, and the genetic composition of populations inside and outside refuges is required to assess the likely consequences of selective hunting for population genetics both inside and outside protected areas. For selectively harvested species, the timing of dispersal events and survival to breeding are therefore critical variables to consider in models quantifying how refuges may buffer the selective effects of harvest.

## Data accessibility

Data for this study are available at Dryad, doi: 10.5061/dryad.f4f6b.
